# Factors Affecting Compliance to Intravitreal Anti-Vascular Endothelial Growth Factor Therapy in Patients with Age-Related Macular Degeneration

**DOI:** 10.4274/tjo.28003

**Published:** 2017-08-15

**Authors:** Onur Polat, Sibel İnan, Serkan Özcan, Mustafa Doğan, Tuncay Küsbeci, Güliz Fatma Yavaş, Ümit Übeyt İnan

**Affiliations:** 1 Afyonkarahisar State Hospital, Ophthalmology Clinic, Afyonkarahisar, Turkey; 2 Afyon Kocatepe University Faculty of Medicine, Department of Ophthalmology, Afyonkarahisar, Turkey; 3 Bozyaka Training and Research Hospital, Ophthalmology Clinic, İzmir, Turkey; 4 Hacettepe University Faculty of Medicine, Department of Ophthalmology, Ankara, Turkey

**Keywords:** Patient compliance, intravitreal injection, ranibizumab, treatment, age-related macular degeneration

## Abstract

**Objectives:**

To determine factors influencing compliance in patients with neovascular age-related macular degeneration (n-AMD) undergoing intravitreal anti-vascular endothelial growth factor (VEGF) therapy.

**Materials and Methods:**

The files of n-AMD patients recommended treatment with ranibizumab were reviewed retrospectively. The treatment regimen was 3 consecutive monthly injections followed by monthly follow-up with intravitreal injections as needed (pro re nata, PRN). Demographic and ocular characteristics were recorded. The patients were categorized into 2 groups: full compliance to treatment, or incomplete loading schedule and/or irregular maintenance treatment. All patients were interviewed by phone about factors affecting continuation of treatment.

**Results:**

Mean age of the 314 patients (160 female, 154 male) included in the study was 71.6±9.1 years. A total of 246 patients (78.3%) could complete 3 consecutive injections at 1-month intervals after the start of treatment; 57 patients (18.2%) did not attend monthly follow-up during the 1-year follow-up period following the 3 consecutive monthly injections. Overall, 39.8% of the patients were not able to fully comply with the ranibizumab treatment by PRN regimen for 1 year. Better visual acuity at baseline, smaller lesion size, living closer to the hospital, higher education and sociocultural level, and better financial status were determined as factors affecting patient compliance. The most frequent reasons to discontinue treatment were fear of injection, disbelief in the benefit of the treatment, financial limitations, continuation of treatment at another center, and comorbid systemic diseases.

**Conclusion:**

Patient compliance and success rates of anti-VEGF therapy may be increased by determining the factors affecting patient compliance and raising awareness about n-AMD among patients and their relatives.

## INTRODUCTION

Age-related macular degeneration (AMD) is a progressive and degenerative disorder of the retinal pigment epithelium, Bruch’s membrane, and choriocapillaris. It is the most common cause of central vision loss in people 65 years and older.^1^ The incidence of AMD is increasing due to the growing elderly population, especially in developed societies, and this constitutes an important health problem today.^2^ The wet (neovascular/exudative) form of AMD can cause rapid loss of useful vision, negatively affecting patients’ daily lives and ability to meet their needs. Therefore, research regarding the management of this type of AMD has been extensive and is ongoing.^3,4,5,6^

The main underlying factor in the pathogenesis of wet AMD is the formation of new vessels in the choroid layer.^3^ The introduction of vascular endothelial growth factor (VEGF) inhibitor therapy into clinical practice has dramatically altered the prognosis of wet AMD. Randomized clinical trials on AMD demonstrate a significant improvement in patients treated with ranibizumab compared to the placebo group, and ranibizumab therapy is recommended monthly to achieve the most effective visual outcomes.^4,5,6^ However, as an alternative to monthly injections, many retina clinics prefer a regimen of 3 monthly intravitreal injections as a loading dose, followed by injections administered as necessary based on visual acuity, optical coherence tomography (OCT), and fundus fluorescein angiography (FFA) findings, because this approach is more feasible in clinical practice.^7,8,9,10,11,12,13^ Having to go to the hospital every month for injection or follow-up, depending on their clinical condition, negatively influences patient compliance for various reasons, therefore affecting the success and outcome of treatment.

The aim of this study was to determine patients’ treatment compliance rates and the factors that affect compliance with treatment and follow-up in patients diagnosed with wet AMD and recommended for ranibizumab therapy.

## MATERIALS AND METHODS

The medical records of patients examined and diagnosed with wet AMD between February 2009 and February 2012 were analyzed retrospectively. A total of 314 patients who were recommended intravitreal ranibizumab injection (IVRI) therapy and who provided consent after being informed of the treatment regimen and duration were included in the study. The study was conducted in accordance with the Declaration of Helsinki and was approved by the Afyon Kocatepe University, Ethics Committee for Clinical Trials (2013/4 Decision: 42). Informed consent forms were obtained from all patients.

The patients were diagnosed with AMD based on clinical, OCT, and FFA findings. Best corrected visual acuity (BCVA) was assessed using the Early Treatment Diabetic Retinopathy Study chart. All patients were informed that the first 3 injections were requisite, after which they would be examined monthly and injections would be repeated as necessary. At the follow-up appointments, all patients underwent ophthalmologic examination and OCT imaging, while FFA was performed only when needed. The treatment regimen consisted of 3 consecutive monthly injections followed by additional injections given when deemed necessary according to OCT and visual acuity findings.

Patients’ demographic features and ocular characteristics were recorded from their records. We determined the number of patients who continued treatment and follow-up appointments for 1 year from the time of diagnosis and investigated the reasons for unsuccessful treatment and follow-up. All patients included in the study were contacted by phone and they or their relatives were asked predetermined questions in order to identify factors that may affect treatment compliance (Table 1). Patients who could not be reached by phone or whose medical records could not be accessed were not included in the study.

Patients were studied in 2 groups according to their compliance to IVRI treatment and the follow-up appointments for 1 year. Patients who regularly received 3 consecutive IVRI treatments after being diagnosed with wet AMD and were followed regularly for 1 year thereafter comprised the ‘compliant group’ (Group 1), while patients who did not regularly receive 3 consecutive IVRI treatments or could not be followed regularly for 1 year comprised the ‘noncompliant group’ (Group 2). Group 2 was further divided into 2 subgroups: patients who completed 3 consecutive months of IVRI treatment but who were unable to complete 1 year of follow-up and treatment (Group 2a), and patients who were unsuccessful in completing their 1 year of follow-up and treatment, including the initial 3 consecutive monthly IVRI injections (Group 2b).

### Statistical Analysis

The data obtained were recorded and analyzed using a statistics software package (SPSS for Windows, version 18.0, SPSS, Chicago, IL, USA). Because the data pertaining to factors affecting patient compliance were qualitative and grouped, chi-square test and chi-square automatic interaction detector (CHAID) analysis were used. P values below 0.05 were considered significant. The correlation between compliance and factors that may affect compliance was assessed using Cramer’s V analysis.

## RESULTS

**Figure 1:**
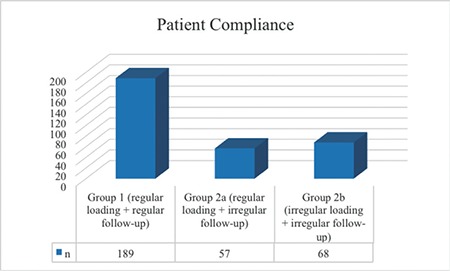
Distribution of patients by study group

**Figure 2:**
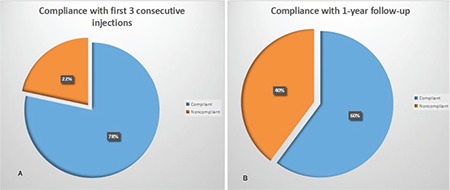
Rates of compliance with the first three consecutive ranibizumab injections (A). Compliance rates of the 57 patients who were unable to comply with the 1-year treatment and follow-up period despite complying with the first 3 injections (B)

The mean age of the 314 patients included in the study was 71.6±9.1 years; the study group comprised 160 female patients and 154 male patients. The number of patients who successfully completed 3 consecutive doses of IVRI treatment and were followed regularly for 1 year (Group 1) was 189 (60.2%), while 125 (39.8%) patients showed inadequate compliance to treatment and follow-up (Group 2). Subgroup analysis of Group 2 showed that 57 patients (18.2%) completed 3 consecutive months of IVRI treatment following diagnosis but did not complete 1 year of follow-up (Group 2a), while 68 patients (21.6%) were not able to comply with follow-up and treatments including the initial 3 consecutive months of IVRI treatment (Group 2b). According to this, 246 patients (78.3%) completed 3 consecutive months of regular IVRI treatment. However, 57 (18.2%) of these patients were not able to regularly attend follow-up after the 3 consecutive IVRI treatments. The number of patients who were able to fully comply with their treatment and follow-up was 189 (60.2%) (Figures 1 and 2).

In the correlation analysis of data obtained from responses provided by patients and/or their relatives during the phone interview, statistically significant relationships emerged between patient compliance with the 1 year follow-up and IVRI treatment and an increase or decrease in the visual acuity of patients after treatment, the visual acuity in the affected eye at the time of diagnosis, how far from the hospital they lived, education level, sociocultural status, age, economic status, fear of injection, the size of the choroid neovascularization lesion detected with FFA, and retirement status (p<0.05). No statistically significant correlations were found between compliance with the 1 year follow-up and IVRI treatment and whether the patients were newly diagnosed or diagnosed in the past, whether they were previously diagnosed with or treated for age-related macular degeneration, the visual acuity level in the fellow eye at the time of diagnosis, the side and number of eyes involved, or patients’ age and employment status (Table 2).

From the results of CHAID analysis, it was determined that the patients’ treatment response in terms of visual acuity was the factor that may have the greatest effect on patient compliance with the 1 year follow-up and treatment. Compliance rates were high among patients whose visual acuity values increased or decreased following treatment, while visual acuity alone showed no positive effect on compliance when it remained unchanged (Table 3). In the second step of the CHAID analysis, it was determined that visual acuity at the time of diagnosis was the most influential factor in compliance among the patients whose visual acuity increased or decreased following treatment. In this group, patients with visual acuity values of 20/40 and higher at time of diagnosis had the highest compliance rates (Table 4). In the third step of the CHAID analysis, the factor that affected compliance in patients whose visual acuity was 20/40 and better was found to be the patients’ place of residence. As the distance between patients’ residence and the treatment center decreased, compliance rates increased.

**Figure 3:**
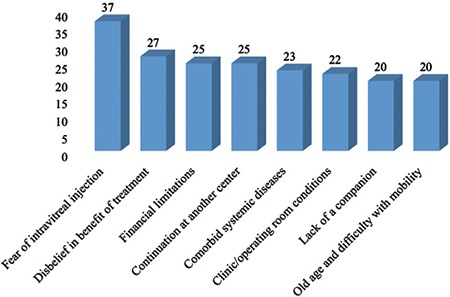
Reasons stated by patients for their lack of compliance

The patients provided between 1 and 4 reasons for not complying with the recommended IVRI treatment and 1 year follow-up. Among the 232 reasons stated by the 125 patients who failed to comply with follow-up and treatment, the most common was a fear of intravitreal injection (29.6%). This was followed by disbelief that treatment would be beneficial/resignation to one’s fate (21.6%), financial difficulty (20.8%), residing in another province or continuing with treatment in another province (20%), comorbid systemic diseases (18.4%), dissatisfaction with the outpatient clinic or operating room conditions (17.6%), lack of relatives to help the patient come to the hospital or not having enough time (16%), and difficulty in coming and going due to old age (16%) (Figure 3).

When we examined the less common reasons, we found that 7 patients (5.6%) stopped attending follow-up because of improved vision and reduced visual complaints after IVRI treatment, but 5 of those patients presented to our clinic again after an average of 6 months due to deteriorating vision and increased visual complaints. Six patients (4.8%) were lost to follow-up due to mortality. The cause of death was cerebrovascular events in 3 patients, heart attack in 2 patients, and traffic accident for 1 patient. Of the patients who were unable to appear regularly to follow-up appointments, 6 (4.8%) were unable to find time due to their job, 5 (4%) were unable to find time due to caring for their spouse who was ill or bedbound, 4 (3.2%) had had a traffic accident a short time before the appointment date, and 3 (2.4%) were unable to come due to severe winter weather conditions.

## DISCUSSION

In this study, we observed that a significant portion of wet AMD patients did not comply adequately with the AMD treatment and follow-up protocol. The factors which had the greatest influence on whether patients continued their treatment were visual acuity change with treatment, visual acuity at the time of diagnosis, and distance to the treatment center.

Controlling angiogenesis with anti-VEGF therapy, which is now the standard treatment modality for wet AMD patients, prevents further macular damage by inhibiting the growth of new vessels and thus stabilizes vision. However, since the underlying pathology continues, anti-VEGF injections need to be continued repeatedly to control angiogenesis. Clinical trials have reported the long-term outcomes of patients complying with the study protocol, and successful treatment outcomes have been achieved.^4,5,6^ Unlike the monthly injections given in clinical trials, however, a regimen of monthly follow-up examinations and additional injections applied as necessary after the first 3 injections is more common in clinical practice worldwide. Some regimens use change in visual acuity during follow-up as the criterion for needing additional injections, while other clinics use the stabilization criterion. According to the stabilization approach, treatment is suspended if vision and anatomy have not shown further improvement in the last 3 follow-up visits, and is reinitiated and continued according to the stabilization criterion in the event of recurrence.^7,8,9,10,11,12,13^ Because clinical practice requires patients to continue attending monthly follow-up, treatment compliance rates may vary depending on factors that push the limits of patients’ compliance with treatment and patients’ level of awareness in terms of the disease and its treatment. While anti-VEGF therapy is promising for AMD patients, the need for repeated intraocular injections makes it difficult to successfully implement. This affects the efficacy and outcomes of treatment.

It has not been adequately addressed in the literature whether or not patients adequately comply with the intravitreal anti-VEGF treatment protocol in wet AMD due to the frequent and repeated injections. As far as we can determine, this subject has not yet been studied in our country, and the few studies conducted abroad have focused more on the reasons why patients discontinue treatment. Droege et al.^14^ investigated the factors and problems affecting compliance with anti-VEGF treatment in AMD in real-life conditions and found a compliance rate of 81.1%. Reasons reported for inability to continue follow-up and treatment included not benefiting from treatment, severe comorbid systemic conditions, continuing treatment at another center, refusing treatment, and death. In addition, similar to our study, distance to the hospital and the necessity for a companion were found to be among the factors that made compliance difficult for patients. In another study with a similar purpose, compliance rates were reported as over 90% in the stabilization phase of treatment and 63.2% in the maintenance phase. Patient compliance was found to depend on the duration of treatment, visual acuity in the fellow eye, and functional outcomes of the initial treatment administered to the affected eye.^15^ In their study investigating the reasons for discontinuing IVRI, Vaze et al.^16^ found that 42.3% of the patients did not continue treatment for various reasons including their doctor ending treatment, frequent visits, difficulty of attendance and follow-up, financial limitations, pain, disbelief in the benefit of the treatment, and refusal of continuance of treatment due to comorbid systemic conditions. Other reasons given were continuing treatment at another center and being unable to continue treatment due to death.

In addition to factors that may affect patients’ compliance with intravitreal injection therapy and follow-up, some studies on the efficacy of intravitreal injection therapy have investigated reasons why some patients terminate treatment as a subtopic.^8,17,18^ These studies determined rates of discontinuing treatment or follow-up to be 4.2%, 8.1%, and 14.2%. When other studies are also considered, the rates of noncompliance with or discontinuation of treatment range between 4.2% and 42.3% in the literature. The differences in compliance rates between studies may be due to differences in the social and financial means and sociocultural levels of the patient populations and/or differences in methodology and follow-up duration between studies.

Unlike in other studies, in the present study we attempted to investigate all factors that may have a positive or negative effect on compliance with IVRI treatment by examining not only the characteristics of patients who either discontinued treatment or did not comply adequately, but also the characteristics of patients who were adequately compliant with treatment and follow-ups, in order to identify solutions for increasing patient compliance.

While patients’ financial means, education level, sociocultural values, disease awareness, and access to treatment are better in developed countries compared to developing or undeveloped countries, the tendency to live alone in old age is more common in developed countries due to the nature of society. This may cause the factors influencing patient compliance to differ from country to country. Health workers, especially ophthalmologists, patients themselves and their relatives, and the authorities that govern health policies all share the great responsibility of improving factors that may affect patient compliance. For example, fear of injection and not benefiting as expected from treatment were found to be the most common reasons stated by patients in our study for not complying adequately with the treatment and 1 year follow-up. This highlights the responsibility of ophthalmologists to properly inform patients about the pathogenesis, course, and treatment of AMD and what treatment responses they should expect.

In Turkey, patients with health insurance have to pay a certain portion of the price of ranibizumab. In addition to this, high transportation costs can be a serious problem for patients and their families whose financial means are limited. Due to the large numbers of patients, the amount of time allotted to patients in outpatient clinics and operating rooms is minimized. Major steps must be taken in the development of policies to ensure that these conditions positively affect patients’ compliance with follow-up and treatment.

Due to its nature, AMD usually emerges in very old patients. Many patients with AMD have a comorbid systemic disease. In our study, 74.3% of the patients had comorbid conditions. In terms of compliance, systemic disease was present in 73.7% of the noncompliant patients and 74.6% of the compliant patients. Although this is not a statistically significant difference, it shows that comorbid systemic disease is the reason that a substantial proportion of patients in this age group were unable to comply adequately. Here again, important responsibility falls on ophthalmologists in terms of explaining every aspect of AMD to patients, and on family physicians in terms of informing patients that postponing their eye examinations may lead to a serious threat to their visual acuity or cause them to miss the effective window for treating AMD.

It is noteworthy that an increase or decrease in final BCVA in the affected eye following treatment emerged in statistical analysis as the most important factor affecting patient compliance. The positive aspect of this result may show that visual success is better in patients with a high level of compliance to treatment, which is expected. However, a decrease in the final BCVA has a positive effect on a patient’s compliance with follow-up and treatment because it evokes the fear of possibly losing one’s vision. Compliance is a necessary but not sufficient condition for successful visual outcomes. Hence, we found a group of patients in our study who did not comply adequately despite their visual acuity having improved with treatment. There may also be factors that make visual success difficult despite adequate compliance, such as disease severity and resistance. In these cases, it is clear that raising the patient’s level of awareness should increase patient compliance. BCVA in the affected eye at time of diagnosis emerged as another important factor and was inversely proportional to compliance, indicating that patients with lower vision at the time of diagnosis were more compliant with treatment and monthly follow-up. This may also have been a result of fear of vision loss. Studies including larger case series that investigate all factors and reasons for noncompliance, in addition to those analyzed in this study, may help to increase patients’ rate of compliance with intravitreal injection therapy.

The limitations of our study are its retrospective design and the subjective nature of the answers to the questions asked by phone. It is possible that the answers were incomplete or biased.

## CONCLUSION

In conclusion, although acceptance of and compliance with treatment seem to be relatively high initially in patients with wet AMD recommended for IVRI therapy, a significant proportion of these patients are unable to fully comply with the treatment regimen within the 1-year follow-up period due to the stated reasons. However, in the time period our study analyzes, intravitreal injection therapy was newly becoming common among AMD patients. The behavioral characteristics of patients with regard to compliance with treatment may have changed in subsequent years. A new study is currently being conducted in our clinic investigating how patient behavior has changed in later years. Determining the factors that may affect treatment compliance in wet AMD patients and raising the awareness of patients and their relatives may facilitate the improvement of treatment compliance and success rates.

## Figures and Tables

**Table 1:**
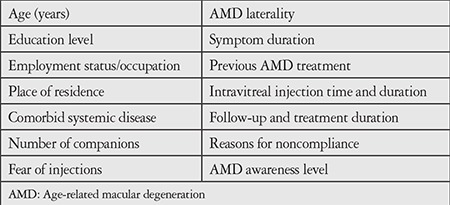
Information collected from the patients

**Table 2:**
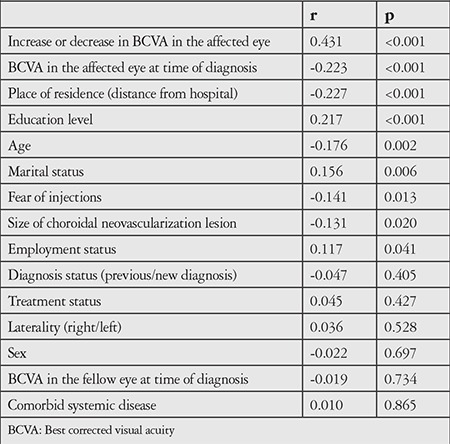
Correlation analysis of factors that may affect patient compliance

**Table 3:**
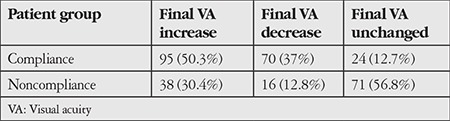
Patient distribution by final visual acuity

**Table 4:**
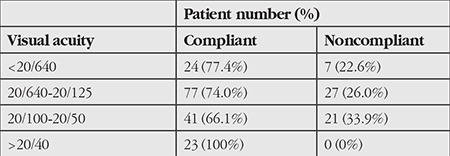
Compliance rates of patients whose best corrected visual acuity increased or decreased during treatment and follow-up, based on initial best corrected visual acuity
